# High-Efficiency Luminescence of Mn^2+^-Doped Two-Dimensional Hybrid Metal Halides and X-Ray Detection

**DOI:** 10.3390/nano15100713

**Published:** 2025-05-09

**Authors:** Yue Fan, Yingyun Wang, Yunlong Bai, Bingsuo Zou, Ruosheng Zeng

**Affiliations:** School of Physical Science and Technology, State Key Laboratory of Featured Metal Materials and Life-Cycle Safety for Composite Structures, Guangxi Key Laboratory of Processing for Non-Ferrous Metals and Featured Materials, Guangxi University, Nanning 530004, China; fyuems@163.com (Y.F.); yunlong_gxu@163.com (Y.B.); zoubs@gxu.edu.cn (B.Z.)

**Keywords:** Mn^2+^-doping, 2D metal halide perovskites, energy transfer, orange-red emission, X-ray imaging

## Abstract

Mn^2+^ doping in metal halide perovskites enables host-to-dopant energy transfer, creating new emission pathways for optoelectronic applications. However, achieving high-efficiency luminescence in 2D systems remains challenging. We synthesized Mn^2+^-doped 2D PEA_2_CdCl_4_ via the hydrothermal method, characterizing its properties through PL spectroscopy, quantum yield measurements, and DFT calculations. Flexible films were fabricated using PDMS and PMMA matrices. The 15% Mn^2+^-doped crystal showed orange–red emission with 90.85% PLQY, attributed to efficient host-to-Mn^2+^ energy transfer and ^4^T_1_→^6^A_1_ transition. Prototype LEDs exhibited stable emission, while PDMS films demonstrated flexibility and PMMA films showed excellent X-ray imaging capability. This work demonstrates Mn^2+^ doping as an effective strategy to enhance luminescence in 2D perovskites, with potential applications in flexible optoelectronics and X-ray scintillators.

## 1. Introduction

Organic–inorganic hybrid metal halides (OIHMH) exhibit excellent optoelectronic properties due to their soft lattice and have broad application prospects in many fields [1,2,3,4,5,6,7,8,9,10,11,12]. Soft lattices refer to crystalline frameworks with high phonon anharmonicity, where weak intermolecular forces (e.g., hydrogen bonds, van der Waals) enable structural flexibility under stimuli. Thanks to the rich diversity of organic materials, various combinations can be achieved between organic and inorganic components, resulting in a range of optoelectronic properties. As a result, OIHMHs have found widespread applications in optoelectronic devices, anti-counterfeiting technology, and information decryption. Among them, two-dimensional organic–inorganic hybrid metal halides are widely used in optoelectronic devices due to their excellent carrier mobility, high light absorption capacity, and photoelectric conversion rate [13,14]. These optical properties are closely related to the unique combination of organic and inorganic components [15]. Therefore, it is an effective optimization method to adjust and improve the optical properties of cadmium-based hybrid metal halides by changing organic or inorganic components. For example, metal halides are modified by adjusting the size of the A-site organic component. The softening of metal halides by organic components will cause instantaneous defects in the excited state, and strong electron–phonon coupling leads to large Stokes shifts and broadband emission [16]. Another method is to change the luminescence color or improve the luminescence efficiency of halides by doping metal ions containing lone pair electrons (such as Pb^2+^, Sn^2+^, Ge^2+^, Sb^3+^, and Bi^3+^) or transition metal ions with d electrons (such as Mn^2+^, Cu^2+^ and Zn^2+^). Therefore, it is necessary to explore a general method to improve the optical properties of cadmium-based metal halides by ion doping.

From a structural point of view, different types of hybrid halides largely determine the observed unusual physical properties [17]. The structural type of the two-dimensional hybrid material depends on the charge of the organic cations between the layers. For the two-dimensional Ruddlesden–Popper (R-P)-phase hybrid perovskite of A’_2_A_n−1_B_n_X_3n+1_, where A’ and A are organic cation with different sizes, B is a metal ion, and X is a halogen anion [18]. Each inorganic metal layer is displaced by an octahedral [BX_6_]^4−^, showing in-plane displacement. The coupled organic bilayers effectively isolate the inorganic sublattice layer, and the reduction in interaction makes it easier for two-dimensional materials to produce exciton emission directly caused by single-layer spatial electron confinement (n = 1).

The polar structure of the R-P phase and the resulting structural distortion provide a rotary knob for adjusting the electronic structure, thereby affecting the optical properties [19]. However, there are few studies on hybrid R-P-phase Cd-based metal halides. According to previous studies, Mn^2+^ is an important photoactive luminescent ion [20,21]. Its unique 3d^5^ electronic configuration endows Mn^2+^ ions with a rich energy level structure and strong spin–orbit coupling, which can produce distinct characteristic luminescence under ultraviolet excitation, especially in the red and near-infrared regions. Moreover, Mn^2+^ ions can effectively enhance the energy transfer efficiency from the host-bound exciton to the d electron of Mn^2+^ ions. This greatly enhances the energy conversion efficiency and reduces the non-radiative recombination loss. Therefore, exploring Mn^2+^-doped Cd-based hybrid metal halides is of great significance. Its unique 3d^5^ electronic configuration endows Mn^2+^ ions with rich energy level structure and strong spin–orbit coupling, which can produce distinct characteristic luminescence under ultraviolet excitation. In addition, Mn^2+^ ions can effectively improve the energy transfer efficiency from the host-bound exciton to the d-orbital of Mn^2+^ ions. This greatly enhances the energy conversion efficiency and reduces the non-radiative recombination loss [22]. Therefore, it is very meaningful to explore an effective way to activate cadmium-based hybrid metal halides. However, PEA_2_CdCl_4_ is susceptible to degradation under humidity and light conditions, leading to reduced reliability in long-term use. Meanwhile, its low photoluminescence (PL) efficiency due to material defects and insufficient excited state dynamics limits its application in optoelectronic devices such as solar cells and LEDs. PEA_2_CdCl_4_, as a material based on an R-P structure [23], possesses good doping and modification potential. Through rational doping and surface modification, its luminescence performance can be significantly improved and its application in the field of optoelectronics can be expanded. Doping with Mn^2+^ ions can significantly improve the photovoltaic performance and stability of PEA_2_CdCl_4_ and enhance its practical application prospects.

In this work, the orthorhombic-phase PEA_2_CdCl_4_ crystal was synthesized by a typical hydrothermal method, and the optical properties of the two-dimensional R-P-phase metal halide PEA_2_CdCl_4_ were further enhanced by appropriate Mn^2+^ ions doping. The Mn^2+^:PEA_2_CdCl_4_ prepared by this method exhibits a red emission at 616 nm under 269 nm excitation, the full width at half maximum (FWHM) is 82 nm, and the optimal doping concentration is 15%. Temperature-dependent emission spectra reveal the obvious activation of Mn^2+^ ions and the small activation energy (Δ*E*_a_ = 10.38 meV), which proves the existence of an effective energy transfer channel from the host to the Mn^2+^ ions. The crystal structure of the Mn^2+^:PEA_2_CdCl_4_ crystal remains unchanged after exposure to air for 2 months. This study uses Mn^2+^ doping to optimize the material, change the mechanism of energy transfer, improve the quantum efficiency, and achieve luminescence modulation, which provides a reference for improving the luminescence performance.

## 2. Materials and Methods

2-Phenylethylamine hydrochloride ((C_6_H_5_NH_2_)CH_2_CH_3_·HCl, 98%), manganese chloride tetrahydrate (MnCl_2_·4H_2_O, 99.9%) and anhydrous cadmium chloride (CdCl_2_, 99%) were purchased from Macklin Inc. (Shanghai, China). Hydrochloric acid (HCl, AR, 37%) was purchased from Aladdin Scientific Inc. (Shanghai, China). All of these chemical agents were used as received without further purification. Firstly, a certain concentration of Mn^2+^ ion doped precursor solution was prepared. Mn^2+^ precursor solution with a molar concentration of 0.1 mmol/mL was obtained by dissolving 1 mmol MnCl_2_·4H_2_O in 10 mL HCl. Subsequently, 0.5 mmol 2-phenylethylamine hydrochloride, 0.25 mmol total metal salts (a mixture of CdCl_2_ and varying volumes of Mn^2+^ precursor solution while maintaining the total Cd^2+^+Mn^2+^ content at 0.25 mmol), and 3 mL HCl were loaded into a 25 mL polytetrafluoroethylene-lined autoclave, which was then sealed in a stainless steel reactor. The reaction proceeded at 180 °C for 150 min followed by natural cooling to room temperature. The products were collected by centrifugation and washed with absolute ethanol, ultimately yielding PEA_2_CdCl_4_ single crystals with varying Mn^2+^ doping concentrations.

## 3. Discussion

**Figure 1:**
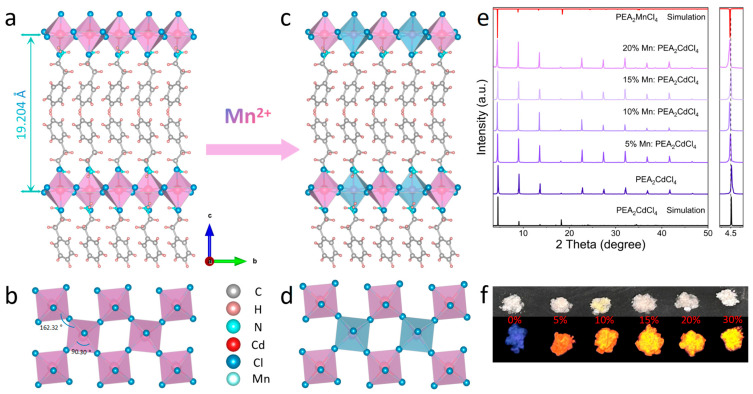
(**a**,**b**) Schematic diagram of crystal structure and corner-shared inorganic sublattice layer of PEA_2_CdCl_4_. (**c**,**d**) Schematic diagram of crystal structure and corner-shared inorganic sublattice layer of Mn^2+^:PEA_2_CdCl_4_. (**e**) PXRD patterns of PEA_2_CdCl_4_ and *x*%Mn^2+^:PEA_2_CdCl_4_. (**f**) The digital photos of *x*%Mn^2+^:PEA_2_CdCl_4_ (*x* = 0–30) under visible light and UV light.

A series of *x*% Mn-doped PEA_2_CdCl_4_ (*x* = 0–20) hybrid metal halides were prepared by the hydrothermal method. As shown in Figure 1a, the crystal structure of the single-layer R-P phase metal halide PEA_2_CdCl_4_ is orthorhombic, and the space group is *Pbca* [23]. In this structure, each Cd atom is coordinated with six Cl atoms to form a [CdCl_6_]^4−^ octahedron. As depicted in Figure 1b, along the c-axis, the inorganic sublattice [CdCl_6_]^4−^ in the compound exhibits angular sharing, and the average bond lengths of Cd_1_–Cl_1_, Cd_1_–Cl_2_ and Cd_1_–Cl_3_ are 2.6497 Å, 2.6513 Å and 2.5550 Å, respectively. The axial compression of [CdCl_6_]^4−^ octahedron leads to its distortion (Figure S1). The degree of octahedral distortion can be quantified using the formula [24,25,26,27]

.
(1) $$\lambda_{\mathrm{oct}}=\frac{1}{6}\sum_{i=0}^{6}{\left[\frac{d_{i}-d_{0}}{d_{0}}\right]}^{2}$$

where *d*_i_ is the Cd-Cl bond length, and *d*_0_ is the average bond length of Cd–Cl in [CdCl_6_]^2−^ octahedron. The result is *λ*_oct_ = 2.96 × 10^−4^. The organic cation PEA^+^ occupies the corner-sharing octahedral gap and the two cations between the layers are bound to each other by van der Waals forces. The organic bimolecular layer and the inorganic layer are alternately arranged to form a two-dimensional structure, and the crystal layer spacing is 19.204 Å. In addition, Figure 1e illustrates the powder XRD spectra of *x*% Mn^2+^:PEA_2_CdCl_4_ (*x* = 0–30) samples. The XRD pattern of the undoped sample (*x* = 0) matches well with the standard card of PEA_2_CdCl_4_, indicating that the sample has no excess impurity phase and exhibits good phase purity. The XRD patterns of different concentrations of Mn^2+^ ions showed that a series of doped samples were successfully synthesized, and the doping of Mn^2+^ did not change the structure of PEA_2_CdCl_4_. The amplification diagram on the right side shows that Mn^2+^ doping has a slight shift on the XRD diffraction peak, which is due to the different ionic radius of Mn^2+^ and Cd^2+^. Figure 1f shows the photo of *x*% Mn^2+^:PEA_2_CdCl_4_ (*x* = 0–30) excited by natural light and ultraviolet light (254 nm). The samples appear as white flakes under natural light irradiation, and under ultraviolet light excitation, there is almost no emission from the undoped samples. However, the Mn^2+^-doped PEA_2_CdCl_4_ exhibit bright red emission, which is spin-forbidden [26,28,29].

To further verify the doping effect of Mn^2+^, scanning electron microscopy (SEM) and X-ray photoelectron spectroscopy (XPS) were used to analyze the morphology, element distribution, and valence state of the doped samples. As shown in Figure S2a, SEM images reveal that the Mn^2+^:PEA_2_CdCl_4_ crystal has a lamellar structure and good crystallinity. Meanwhile, the energy dispersive spectroscopy (EDS) analysis (Figure S2b–d) also confirms the presence of N, Cd, Mn and Cl, and the elements are evenly distributed. This further supports the phase purity of the PEA_2_CdCl_4_ and the successful doping of Mn^2+^ ions. Subsequently, XPS was used to analyze the elemental valence state and chemical composition of the samples. The XPS spectrum of Figure S3a also shows the presence of N, Cd, Mn and Cl elements. The high-resolution XPS spectrum shown in Figure S3b reveals distinct characteristic peaks at 199 and 198 eV, which belong to 2p_3/2_ of Cl, respectively. The characteristic peaks at 412.5 eV and 405.5 eV in Figure S3c correspond to the 3d_3/2_ and 3d_5/2_ orbitals of Cd, respectively. In summary, the elemental compositions of the samples and their valence states were determined. Both Mn^2+^ and Cd^2+^ are present in the +2 valence. The similar valence state and ionic radius also indicate that the addition of Mn^2+^ ions preferentially replaces the sites of Cd^2+^. The above results show that the element distribution of Mn^2+^:PEA_2_CdCl_4_ synthesized by the hydrothermal method is uniform and has high phase purity.

**Figure 2:**
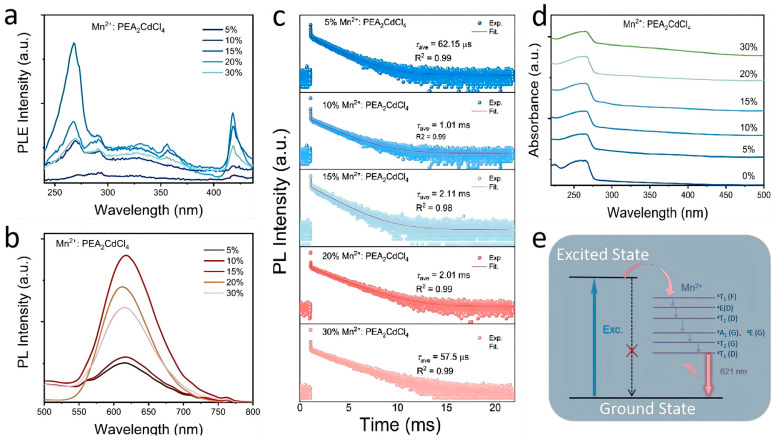
(**a**) PLE spectra of *x*% Mn^2+^-doped PEA_2_CdCl_4_ (*x* = 5–30, λ_em_ = 616 nm). (**b**) PL spectra of *x*% Mn^2+^-doped PEA_2_CdCl_4_ (*x* = 0–30, λ_ex_ = 269 nm). (**c**) Absorption spectra of *x*%Mn^2+^:PEA_2_CdCl_4_. (**d**) The photophysical energy level transition mechanism of Mn^2+^:PEA_2_CdCl_4_. (**e**) PL decay curves of Mn^2+^-doped PEA_2_CdCl_4_ (λ_ex_ = 269 nm, λ_em_ = 616 nm).

It is well known that Mn^2+^ ions, as transition metals with *d* electrons, significantly improve the optical properties of metal halides by doping the crystal lattice. As shown in Figure 1f, the undoped sample exhibits almost no emission under 254 nm excitation. In contrast, after doping with Mn^2+^ ions, the crystal shows obvious red emission. To further investigate the luminescence properties, absorption, photoluminescence excitation (PLE) spectra, photoluminescence (PL) spectra and PL decay curves were performed. Figure 2d illustrates the absorption spectra of the samples doped with different concentrations of Mn^2+^. Compared with the sample with *x* = 0, the absorption band edge shows a slight red shift with the increase in Mn^2+^ doping concentration. A smaller absorption band edge also indicates a larger band gap value. The band gap of *x*% Mn^2+^:PEA_2_CdCl_4_ (*x* = 0, 15, 30) was calculated by the Kubelka–Munk (K-M) Equation (2),

(2) $${\left[F\left(R_{\infty }\right)h\nu \right]}^{n}=A(h\nu -E_{g})$$

where *F*(*R*_∞_) is the Kubelka–Munk (K–M) function, *hυ* is the photon energy, A is the scaling constant, and *E_g_* is the band gap. For direct semiconductors, n = 2 is used to calculate the undoped PEA_2_CdCl_4_ and Mn^2+^ doped samples. As shown in Figure S4, the band gap of undoped PEA_2_CdCl_4_ is 4.44 eV, while the band gaps of 15% and 30% Mn^2+^:PEA_2_CdCl_4_ are 4.37 eV and 4.30 eV, respectively. The band gap decreases with an increasing Mn^2+^ doping concentration, which is consistent with its absorption spectra.

The luminescence properties of Mn^2+^:PEA_2_CdCl_4_ were further investigated by monitoring the optical behaviors, such as PL, PLE, and PL decay curves. As shown in Figure 2a, the performance of Mn^2+^ ions with different doping concentrations in the PLE spectra showed a consistent trend with the corresponding absorption spectra. The five excitation peaks in the PLE spectra are located at 269, 291, 329, 356 and 418 nm, which correspond to ^6^A_1_(S)→^4^A_2_(F), ^6^A_1_(S)→^4^T_1_(F), ^6^A_1_(S)→^4^E(D), ^6^A_1_(S)→^4^T_2_(D) and ^6^A_1_(S)→^4^A_1_, ^4^E_1_(G) of Mn^2+^, respectively [30]. At the same time, with the increase in Mn^2+^ ion doping, the absorption at 269 nm is enhanced, which further confirms that it comes from the absorption of Mn^2+^ ions. As shown in Figure 1f and Figure 2b, the undoped sample does not radiate outward under ultraviolet excitation. After the introduction of Mn^2+^, the sample exhibits a narrow-band emission at 616 nm under 269 nm, and its FWHM is about 78 nm. This indicates that the emission comes from the ^4^T_1_→^6^A_1_(S) transition of Mn^2+^ ions [31], and the emission intensity reaches the peak value when the doping concentration reaches 15%. Simultaneously, the PL intensity of Mn^2+^:PEA_2_CdCl_4_ decreases rapidly at a high doping concentration (after doping concentration exceeds 15%), which is attributed to the concentration quenching effect after Mn^2+^ doping (Figure S5) [32]. Additionally, as shown in Figure S6, the peak position of *x*% Mn^2+^:PEA_2_CdCl_4_ does not shift at different excitations and different Mn^2+^ ion doping concentrations (*x* = 5–30), which further confirms that the orange-red emission arises from the d-d transition of the Mn^2+^ ion spin-forbidden. There is only a single luminescent center. After that, PL attenuation monitoring was performed on samples with different doping concentrations to further exclude the possibility of other emission sources. The PL decay curves of 5–30% Mn^2+^: PEA_2_CdCl_4_ can be fitted by use of the formulas [33]

(3) $$I_{\left(t\right)}=I_{0}+A_{1}\exp\left(t/\tau_{1}\right)+A_{2}\exp\left(t/\tau_{2}\right)+A_{3}exp(t/\tau_{3})$$

(4) $$\tau_{ave}=\left(A_{1}\tau_{1}^{2}+A_{2}\tau_{2}^{2}+A_{3}\tau_{3}^{2}\right)/(A_{1}\tau_{1}+A_{2}\tau_{2}+A_{3}\tau_{3})$$

where *I*_(t)_ is the intensity of the PL at the time of *t*, *I*_(0)_ is the initial intensity, *A*_1_, *A*_2_, and *A*_3_ are constants, and *τ*_1_, *τ*_2_, and *τ*_3_ are the decay times of the corresponding exponential components. The average lifetimes after fitting were 62.15 μs, 1.01 ms, 2.11 ms, 2.01 ms, and 57.5 μs, respectively. Under 269 nm, the PL decay lifetime shows a consistent trend with the emission spectrum (Figure 2c), and the long lifetime (in milliseconds) also confirms that the red narrow-band emission originates from the spin-forbidden transition of Mn^2+^ ions. When the doping concentration exceeds the optimal doping concentration of Mn^2+^ ions (15%), the lifetime also decreases with the increase in doping concentration. Further optical performance tests show that under 254 nm excitation, compound 1 achieves a photoluminescence quantum yield (PLQY) of 90.85% (Figure S7), indicating its great potential for optoelectronic applications.

**Figure 3:**
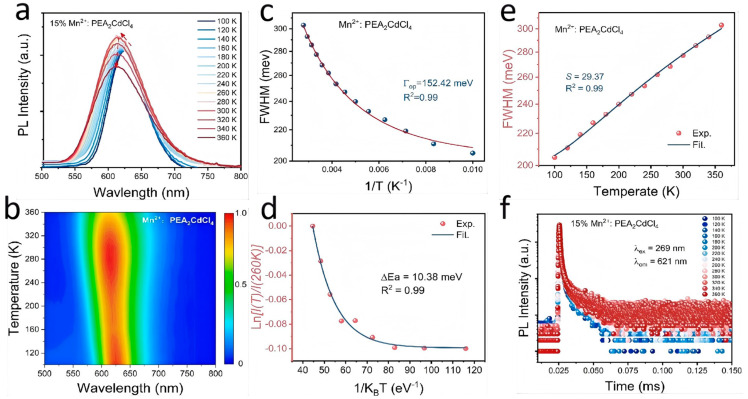
(**a**,**b**) Temperature-dependent PL spectra and pseudo-color map of 15%Mn^2+^:PEA_2_CdCl_4_; (**c**) electron-phonon coupling energy of 15%Mn^2+^:PEA_2_CdCl_4_; (**d**) Huang–Rhys factor *S* of 15%Mn^2+^:PEA_2_CdCl_4_; (**e**) activation energy of 15% Mn^2+^:PEA_2_CdCl_4_; (**f**) PL decay curves of Mn^2+^:PEA_2_CdCl_4_ under different temperatures.

To explore the optical behavior of the material at different temperatures, we monitored the temperature-dependent PL spectra of 15%Mn^2+^:PEA_2_CdCl_4_. Different from the previously studied process of Sb^3+^ ion doping with ns^2^ electrons, Mn^2+^ exhibits different optical behaviors. Figure 3a,b illustrate the temperature-dependent PL spectra and corresponding pseudo-color maps in the temperature range of 100 K to 360 K. From these figures, it can be observed that the PL peak position consistently exhibits a blue shift with increasing temperature, while its intensity first increases and then decreases. As the temperature increases, the intensity of the crystal field due to lattice expansion decreases, which causes the blue shift of the peak position. We can fit the dependence of FWHM on temperature using Formula (5) [33],

(5) $${FWHM}_{\left(T\right)}=2.36\sqrt{S}{\hbar \omega }_{phonon}\sqrt{coth\frac{{\hbar \omega }_{phonon}}{2K_{B}T}}$$

Here, the phonon frequency is the phonon frequency, *S* is the Huang–Rhys factor, and *K_B_* is the Boltzmann constant. The fitted value of the Huang–Rhys factor *S* is about 29.37 (Figure 3e). As a parameter describing electron–phonon interaction, *S* is closely related to the Jahn–Teller distortion. The larger *S* value indicates that there is a strong electron–phonon coupling in Mn^2+^:PEA_2_CdCl_4_. In addition, the electron–phonon coupling energy Γ*_op_* is obtained by fitting the FWHM with 1/T using the Formula (6) [34]

(6) $$\Gamma \left(T\right)=\Gamma_{0}+\Gamma_{op}/\left(e^{\hbar \omega_{op}/K_{B}T}-1\right)$$

Γ_0_ and Γ(T) are the intrinsic half-peak widths at 0 and T K, respectively. Γ_(op)_ is the electron–phonon coupling energy, *ℏω_op_* is the phonon energy and K_B_ is a Boltzmann constant. The fitted Γ*_op_* value is about 152.42 meV (Figure 3c).

Meanwhile, the FWHM increases continuously with increasing temperature, which is mainly attributed to the diffusion of more excited state electrons into the vibrational energy level caused by the temperature increase, which in turn leads to the radiative transition to the ground state. It is worth noting that the PL intensity increases with the increase in temperature in the range of 100–260 K (Figure S8). This is due to the temperature dependence of the PL of Mn^2+^ ions from the vibrational activation of the radiative d-d transition [19,35]. In this process, the sensitization of the d-d transition of Mn^2+^ ions is gradually activated by lattice vibration heat, and the increase in emission intensity can be observed. As shown in Figure 3a, it decreases with the increase in temperature in the range of 280–360 K. This is due to the rise in non-radiative complexation due to electron–phonon coupling and defects with increasing temperature. Then, the thermal activation energy (∆*E_a_*) required for the exciton to transfer from the host to the Mn^2+^-doped ion is estimated using Formula (7):

(7) $$I_{Mn}(T)=I_{0}*e^{{-∆E}_{a}/K_{B}T}$$

*K*_B_ is the Boltzmann constant, *I*_0_ is the initial strength and *I_Mn_(T)* is the integrated PL intensity at different temperatures (100–260 K). In the range of 100–260 K, the thermal activation energy Δ*E*_a_ of Mn^2+^:PEA_2_CdCl_4_ is about 10.38 meV, derived by fitting the dependence of PL intensity on the reciprocal of temperature [36,37]. Such a small potential barrier is also the reason why excitons can be effectively transferred to Mn^2+^ ions, resulting in the maximum PL intensity. Therefore, further heating (i.e., from 260 to 360 K) will reduce the intensity of PL. As shown in Figure 3f, the PL decay curves at different temperatures were monitored to gain a deeper understanding of this unusual phenomenon. The PL decay at different temperatures shows a similar trend to its PL. The results show that the doping of Mn^2+^ ions further opens the channel of energy radiation. After the material is excited by light, the electrons transition from the ground state to the unstable excited state, and then the excitons transfer from the host to the dopant Mn^2+^ downward radiative transition (Figure 2e).

**Figure 4:**
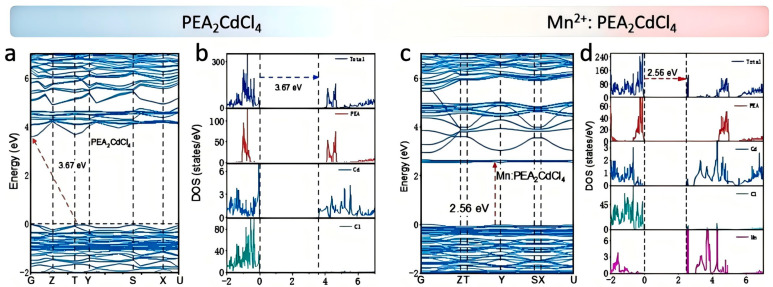
(**a**,**b**) Electronic energy band structures and corresponding electronic structures of PEA_2_CdCl_4_. (**c**,**d**) Electronic energy band structures and corresponding electronic structures of Mn^2+^:PEA_2_CdCl_4_.

The electronic structure and optical band gap of the samples can be explained and predicted by density functional theory (DFT). We calculated the electronic band structure and projected density of states (PDOS) to investigate the influence of Mn^2+^ ion doping on the electronic properties and optical band gap. As shown in Figure 4a, the undoped PEA_2_CdCl_4_ exhibits an indirect band gap of 3.67 eV, so the downward transition radiation process is dipole-forbidden. The doped sample is based on the structure of the undoped sample, with Mn^2+^ replacing Cd^2+^, and the doping concentration is 15%. The optical band gap is reduced to 2.56 eV after doping with Mn^2+^. The discrepancy between the calculated and experimental values can be attributed to the tendency of standard DFT calculations to underestimate the band gap of materials, often resulting in values that are lower than the actual measurements. This phenomenon has been well-documented in the context of organic–inorganic hybrid metal halides. Furthermore, the computed results provide a deeper understanding of the electronic structure. It is well known that the doping of Mn^2+^ will disturb the electronic composition of the host near the optical band gap, mainly due to the introduction of Mn^2+^ into the intermediate energy levels. The PDOS, valence band maximum (CBM) and conduction band minimum (VBM) shown in Figure 4b reveal that the VBM and CBM of undoped PEA_2_CdCl_4_ are composed of Cd and Cl, which are confined to [CdCl_4_]^2−^. In contrast, as shown in Figure 4c,d, the CBM and VBM of Mn:PEA_2_CdCl_4_ are composed of Mn and Cl, respectively.

### Stability and Applications

**Figure 5:**
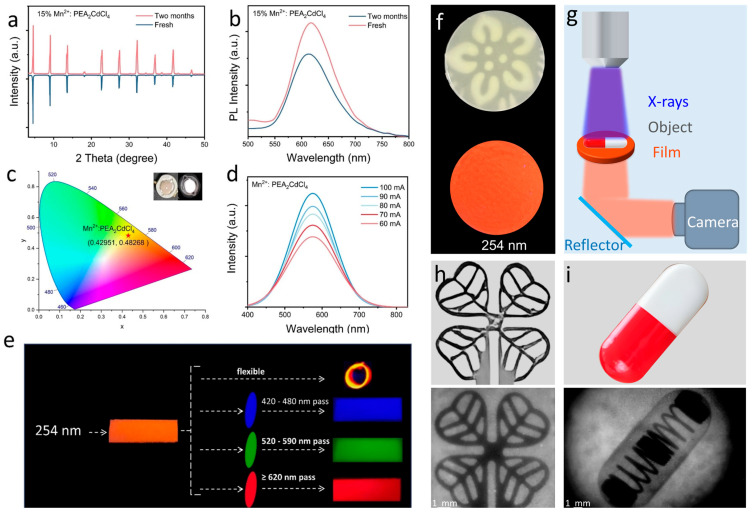
(**a**,**b**) XRD patterns and PL spectra of fresh Mn^2+^:PEA_2_CdCl_4_ and Mn^2+^:PEA_2_CdCl_4_ after being exposed in air; (**c**) CIE coordinates of Mn^2+^:PEA_2_CdCl_4_ based LEDs are (0.42951, 0.48268) (red star). The illustrations show a working photos of the device; (**d**) PL spectra of LED were driven at different currents (60–100 mA). (**e**) The flexible film is based on 15%Mn^2+^:PEA_2_CdCl_4_@PDMS and an information reading scheme in red, green and blue channels by filters. (**f**) Photos of 15%Mn^2+^:PEA_2_CdCl_4_@PMMA film under natural light and UV light. (**g**) Schematic of the indirect X-ray imaging system. (**h**) X-ray imaging of metal pendant. (**i**) Application of X-ray imaging in metal detection.

As shown in Figure 5a, the X-ray diffraction (XRD) patterns of Mn^2+^:PEA_2_CdCl_4_ after two months of exposure to air closely match those of freshly synthesized samples, indicating excellent phase stability with no formation of secondary phases. Simultaneously, we monitored the PL spectra of the samples after exposure to air, as shown in Figure 5b. The PL intensity can be maintained at 74% of the freshly prepared sample. Additionally, Figure 5c shows the LED based on 15%Mn^2+^:PEA_2_CdCl_4_ powder to study its potential utility in practical applications. The illustrations depict the LED at a driving voltage of 3.5 V, producing a stable bright orange light with CIE coordinates of (0.332, 0.199). At different driving currents (60–100 mA), the LED spectrum also shows high color stability (Figure 5d). These results show that Mn^2+^:PEA_2_CdCl_4_ can withstand the heat brought by the operation of the device and has good luminescence stability, making it very suitable for use in solid-state lighting and backlight displays. To further explore the material’s applicability, we mixed 15%Mn^2+^:PEA_2_CdCl_4_ powder with polydimethylsiloxane (PDMS) to create a flexible film, as shown in Figure 5e. We used different narrow-band pass filters (allowing > 620 nm pass, 520–590 nm pass and 420–480 nm pass, respectively) to extract information in the RGB channels. The results yield customized color outputs, demonstrating significant potential for application in the intelligent era. The prepared flexible film exhibits a high degree of flexibility without compromising its luminous efficiency. In addition to polymerization with PDMS, we further prepared highly transparent films by mixing 15% Mn^2+^:PEA_2_CdCl_4_ powder with polymethyl methacrylate (PMMA) (Figure 5f) and demonstrated X-ray imaging using the system shown in Figure 5g. As illustrated in Figure 5h, the 15% Mn^2+^:PEA_2_CdCl_4_@PMMA film enabled the clear imaging of small metal pendants, with leaf contours smaller than 1 mm distinctly visible. Furthermore, the 15% Mn^2+^:PEA_2_CdCl_4_ film also exhibited an excellent detection capability for the spring inside the capsule. These results highlight the great potential use of 15% Mn^2+^:PEA_2_CdCl_4_ as an X-ray scintillator for X-ray imaging applications. Overall, these findings contribute to the development of smart luminescent materials, and highlight their potential in various practical applications.

## 4. Conclusions

In summary, we have designed and synthesized a novel Mn^2+^-doped two-dimensional hybrid metal halide, PEA_2_CdCl_4_, which exhibits intense orange-red emission with an almost 100% PLQY. Experimental and theoretical analyses indicate that this high-efficiency luminescence can be attributed to the effective energy transfer between the host and Mn^2+^, followed by the ^4^T_1_-^6^A_1_ transition in Mn^2+^. Furthermore, we fabricated LEDs based on Mn^2+^:PEA_2_CdCl_4_ powders and flexible X-ray scintillator films, both of which demonstrated excellent optoelectronic performance, highlighting the broad application potential of Mn^2+^:PEA_2_CdCl_4_ in the field of optoelectronics.

## Data Availability

Dataset available on request from the authors.

## Supplementary Materials

The following supporting information can be downloaded at: https://www.mdpi.com/article/10.3390/nano15100713/s1, Experimental methods, related test methods and computational details, SEM and EDS-mapping images, XPS spectra, spectra and PLQY; Figure S1: (a) Schematic diagram of organic amine insertion into inorganic sublattices in PEA_2_CdCl_4_ and Mn^2+^:PEA_2_CdCl_4_ SCs; (b) the molecular formula of organic amine PEA; (c) A schematic diagram of [CdCl_6_]^4−^ octahedrons in PEA_2_CdCl_4_ and [CdCl_6_]^4−^ octahedrons substituted by [MnCl_6_]^4−^ in Mn^2+^:PEA_2_CdCl_4_; Figure S2: (a–d) The SEM image and EDS-mappings of N, Cd, Mn and Cl of 15% Mn^2+^:PEA_2_CdCl_4_. (f) Energy-dispersed spectrum of 15% Mn^2+^:PEA_2_CdCl_4_; Figure S3: (a) XPS spectra of Mn^2+^:PEA_2_CdCl_4_ and 15% Mn^2+^:PEA_2_CdCl_4_. (b,c) High-resolution XPS spectra of Cd 3d and Cl 2p in PEA_2_CdCl_4_ and 15% Mn^2+^:PEA_2_CdCl_4_. (d) High-resolution XPS spectra of Mn 2p in 15% Mn^2+^:PEA_2_CdCl_4_; Figure S4: *x*% Mn^2+^:PEA_2_CdCl_4_ (*x* = 0, 15, 30) band gaps calculated by using the K-M equation; Figure S5: Changes of PL Intensity and peak position with Mn^2+^ doping concentration; Figure S6: PL spectra under different excitations of 15%Mn^2+^:PEA_2_CdCl_4_; Figure S7: PLQY of 15%Mn^2+^:PEA_2_CdCl_4_; Figure S8: Temperature-dependent normalized PL intensity changes of 15%Mn^2+^:PEA_2_CdCl_4_; Figure S9: Schematic diagram of preparation of flexible film.

## References

[1] Zhu M., Qin L., Xia Y., Liang J., Wang Y., Hong D., Tian Y., Tie Z., Jin Z. (2023). Antimony doped CsPbI_2_Br for high-stability all-inorganic perovskite solar cells. Nano Res..

[2] Guo L.F., Wang Z.X., Zhou Y.Q., Huang L., Chen W., Liu Y.C., Wang F., Zhang X., Chen W.W., Lai J. (2024). Unity emission in organic phosphonium antimony halide for high color rendering white LED. J. Lumin..

[3] Lin Z., Wu Y.-N., Xu S.-Y., Chen B.-C., Huang P.-W., Qi X.-H., Lin Y.-P., Du K.-Z. (2024). Dopant effect on the optical and thermal properties of the 2D organic–inorganic hybrid perovskite (HDA)_2_PbBr_4_. Dalton Trans..

[4] Sun C., Guo Y.H., Han S.S., Li J.Z., Jiang K., Dong L.F., Liu Q.L., Yue C.Y., Lei X.W. (2020). Three-Dimensional Cuprous Lead Bromide Framework with Highly Efficient and Stable Blue Photoluminescence Emission. Angew. Chem. Int. Ed..

[5] Sun C., Jiang Y., Cui M., Qiao L., Wei J., Huang Y., Zhang L., He T., Li S., Hsu H.-Y. (2021). High-performance large-area quasi-2D perovskite light-emitting diodes. Nat. Commun..

[6] Wang C., Liu Y., Feng X., Zhou C., Liu Y., Yu X., Zhao G. (2019). Phase Regulation Strategy of Perovskite Nanocrystals from 1D Orthomorphic NH_4_PbI_3_ to 3D Cubic (NH_4_)_0.5_Cs_0.5_Pb(I_0.5_Br_0.5_)_3_ Phase Enhances Photoluminescence. Angew. Chem. Int. Ed..

[7] Wang C., Zhao N., Zhang H., Zhang X., Lin X., Liu H., Dang F., Zhang W., Sun J., Chen P. (2023). Ultrawide UV to NIR Emission in Double Perovskite Nanocrystals via the Self-Trapping State Engineering Strategy. ACS Sustain. Chem. Eng..

[8] Wu S., Liu Y. (2023). Recent advancements and manipulation strategies of colloidal Cs_2_B^I^B^III^X_6_ lead-free halide double perovskite nanocrystals. Nano Res..

[9] Zhang Z., Cheng H., Teng S., Huang K., Wang D., Yang W., Xie R. (2022). Thermally Induced Reversible Fluorescence Switching of Lead Chloride Hybrids for Anticounterfeiting and Encryption. Inorg. Chem..

[10] Zhou G., Jiang X., Molokeev M., Lin Z., Zhao J., Wang J., Xia Z. (2019). Optically Modulated Ultra-Broad-Band Warm White Emission in Mn^2+^-Doped (C_6_H_18_N_2_O_2_)PbBr_4_ Hybrid Metal Halide Phosphor. Chem. Mater..

[11] Zhou G., Xu Y., Xia Z. (2020). Perovskite Multiple Quantum Wells on Layered Materials toward Narrow-Band Green Emission for Backlight Display Applications. ACS Appl. Mater. Interfaces.

[12] Xu H.K., Wei X.F., Zeng H., Jiang C.H., Wang Z.F., Ouyang Y.G., Lu C.Y., Jing Y., Yao S.W., Dai F.N. (2023). Recent progress of two-dimensional metal-organic-frameworks: From synthesis to electrocatalytic oxygen evolution. Nano Res..

[13] Wang M., Zhang X., Liu L., Zhang X., Yan J., Jin W., Zhang P., Wang J. (2024). Stable and Highly Efficient Photocatalysis with Two-Dimensional Organic–Inorganic Hybrid Perovskites. ACS Omega.

[14] Zhou J., Chu Y., Huang J. (2016). Photodetectors Based on Two-Dimensional Layer-Structured Hybrid Lead Iodide Perovskite Semiconductors. ACS Appl. Mater. Interfaces.

[15] Rok M., Starynowicz P., Ciżman A., Zaręba J.K., Piecha-Bisiorek A., Bator G., Jakubas R. (2020). Advances and Property Investigations of an Organic–Inorganic Ferroelectric: (diisopropylammonium)_2_[CdBr_4_]. Inorg. Chem..

[16] Li X., Liu A., Wang Z., Wei Y., Lin Q., Chen Y., Liu Y., Hong M. (2023). Boosting self-trapped exciton emission from Cs_3_Cu_2_I_5_ nanocrystals by doping-enhanced exciton-phonon coupling. Nano Res..

[17] Mao L., Stoumpos C.C., Kanatzidis M.G. (2018). Two-Dimensional Hybrid Halide Perovskites: Principles and Promises. J. Am. Chem. Soc..

[18] Wang Y., Su B., Lin G., Lou H., Wang S., Yue C.-Y., Lei X. (2022). Exploring the Ruddlesden–Popper layered organic–inorganic hybrid semiconducting perovskite for visible-blind ultraviolet photodetection. CrystEngComm.

[19] Race J.T., Liu T., Woodward P.M. (2024). Structure Directing Forces in Hybrid Layered Double Perovskites Containing Aromatic Organic Cations. Cryst. Growth Des..

[20] Guan M., Hao J., Qiu L., Molokeev M.S., Ning L., Dai Z., Li G. (2024). Two-Dimensional Hybrid Perovskite with High-Sensitivity Optical Thermometry Sensors. Inorg. Chem..

[21] Ha S.K., Shcherbakov-Wu W., Powers E.R., Paritmongkol W., Tisdale W.A. (2021). Power-Dependent Photoluminescence Efficiency in Manganese-Doped 2D Hybrid Perovskite Nanoplatelets. ACS Nano.

[22] Yuan X., Ji S., De Siena M.C., Fei L., Zhao Z., Wang Y., Li H., Zhao J., Gamelin D.R. (2017). Photoluminescence Temperature Dependence, Dynamics, and Quantum Efficiencies in Mn^2+^-Doped CsPbCl_3_ Perovskite Nanocrystals with Varied Dopant Concentration. Chem. Mater..

[23] Yue Z.Y., Luo W., Wang N., Li H.K., Xu Z.J., Feng Y., Shi C., Ye H.Y., Miao L.P. (2023). Two-dimensional organic-inorganic hybrid perovskite ferroelastics: (PEA)_2_ CdCl_4_, (3-FPEA)_2_CdCl_4_, and (4-FPEA)_2_ CdCl_4_. CrystEngComm.

[24] Fu P., Quintero M.A., Welton C., Li X., Cucco B., De Siena M.C., Even J., Volonakis G., Kepenekian M., Liu R. (2022). Short Aromatic Diammonium Ions Modulate Distortions in 2D Lead Bromide Perovskites for Tunable White-Light Emission. Chem. Mater..

[25] Jana M.K., Song R., Xie Y., Zhao R., Sercel P.C., Blum V., Mitzi D.B. (2021). Structural descriptor for enhanced spin-splitting in 2D hybrid perovskites. Nat. Commun..

[26] Su B., Molokeev M.S., Xia Z. (2020). Unveiling Mn^2+^ Dopant States in Two-Dimensional Halide Perovskite toward Highly Efficient Photoluminescence. J. Phys. Chem. Lett..

[27] Huang T., Li K., Lei J.Y., Niu Q., Peng H., Zou B.S. (2023). Origin of singlet self-trapped exciton and enhancement of photoluminescence quantum yield of organic-inorganic hybrid antimony(III) chlorides with the (SbCl_5_)^2−^ units. Nano Res..

[28] Han P., Zhang X., Luo C., Zhou W., Yang S., Zhao J., Deng W., Han K. (2020). Manganese-Doped, Lead-Free Double Perovskite Nanocrystals for Bright Orange-Red Emission. ACS Cent. Sci..

[29] Panda D.P., Swain D., Chaudhary M., Mishra S., Bhutani G., De A.K., Waghmare U.V., Sundaresan A. (2022). Electron–Phonon Coupling Mediated Self-Trapped-Exciton Emission and Internal Quantum Confinement in Highly Luminescent Zero-Dimensional (Guanidinium)_6_Mn_3_X_12_ (X = Cl and Br). Inorg. Chem..

[30] Zhang W., Sui P., Zheng W., Li L., Wang S., Huang P., Zhang W., Zhang Q., Yu Y., Chen X. (2023). Pseudo-2D Layered Organic-Inorganic Manganese Bromide with a Near-Unity Photoluminescence Quantum Yield for White Light-Emitting Diode and X-Ray Scintillator. Angew. Chem. Int. Ed..

[31] Liu X.T., Yang J.P., Chen W.Y., Yang F., Chen Y.H., Liang X.J., Pan S., Xiang W.D. (2023). (CH_3_)_4_N_2_Mn_0.6_Zn_0.4_Br_4_: Lead-free MnII-based hybrid halide with high photoluminescence quantum yield for backlight displays. Nano Res..

[32] Kou T., Wei Q., Jia W., Chang T., Peng C., Liang Y., Zou B. (2022). Light Emission Enhancement of (C_3_H_10_N)_4_Pb_1−x_Mn_x_Br_6_ Metal-Halide Powders by the Dielectric Confinement Effect of a Nanosized Water Layer. ACS Appl. Mater. Interfaces.

[33] Huang H., Yang Y., Qiao S., Wu X., Chen Z., Chao Y., Yang K., Guo W., Luo Z., Song X. (2023). Accommodative Organoammonium Cations in A-Sites of Sb—In Halide Perovskite Derivatives for Tailoring BroadBand Photoluminescence with X-Ray Scintillation and White-Light Emission. Adv. Funct. Mater..

[34] Zhang J., Ma Y.-X., Wu M., He Q., Chen S., Ju P., He Y.-C., Lei X. (2024). Zero-dimensional organic–inorganic hybrid zinc halide with stable broadband blue light emissions. CrystEngComm.

[35] Zhou G., Liu Z., Molokeev M.S., Xiao Z., Xia Z., Zhang X.-M. (2021). Manipulation of Cl/Br transmutation in zero-dimensional Mn^2+^-based metal halides toward tunable photoluminescence and thermal quenching behaviors. J. Mater. Chem. C.

[36] Luo B., Guo Y., Li X., Xiao Y., Huang X., Zhang J.Z. (2019). Efficient Trap-Mediated Mn^2+^ Dopant Emission in Two Dimensional Single-Layered Perovskite (CH_3_CH_2_NH_3_)_2_PbBr_4_. J. Phys. Chem. C..

[37] Pinchetti V., Anand A., Akkerman Q.A., Sciacca D., Lorenzon M., Meinardi F., Fanciulli M., Manna L., Brovelli S. (2018). Trap-Mediated Two-Step Sensitization of Manganese Dopants in Perovskite Nanocrystals. ACS Energy Lett..

