# Supplementary material for: High-Efficiency Luminescence of Mn2+-Doped Two-Dimensional Hybrid Metal Halides and X-Ray Detection
Source: Nanomaterials (Basel). 2025 May 9;15(10):713. doi: 10.3390/nano15100713 (PMC12114107; doi:10.3390/nano15100713)
Supplement: Supplementary file 1 [file nanomaterials-15-00713-s001.zip › nanomaterials-3620309-supplementary.pdf]

Supplementary Information

High-Efficiency Luminescence of Mn2+-Doped Two-Dimensional Hybrid Metal Halides and X-Ray Detection

Yue Fan, Yingyun Wang*, Yunlong Bai, Bingsuo Zou and Ruosheng Zeng*

School of Physical Science and Technology, State Key Laboratory of Featured Metal Materials and Life-Cycle Safety for Composite Structures, Guangxi Key Laboratory of Processing for Non-Ferrous Metals and Fea-tured Materials, Guangxi University, Nanning 530004, China

* Corresponding author: dtwyy0729@163.com; zengrsh@guet.edu.cn

Experimental section

Materials

2-Phenylethylamine hydrochloride (($(C_6H_5NH_2)CH_2CH_3$·HCl, 98%), Manganese chloride tetrahydrate ($MnCl_2·4H_2O$, 99.9%) and Anhydrous cadmium chloride ($CdCl_2$, 99%) were purchased Macklin. Hydrochloric acid (HCl, AR, 37%) was purchased from Aladdin. All of these chemical agents were used as received without further purification.

Methods.

Synthesis of 2D PEA_2CdCl_4 and Mn^{2+}:PEA_2CdCl_4 SCs

A series of x%Mn^{2+}:PEA_2CdCl_4 (x = 0, 5, 10, 15, 20 and 30) were prepared by the hydrothermal method.

Synthesis of PEA_2CdCl_4 SCs

Here, 0.5 mmol phenylethylamine hydrochloride, 0.25 mmol $CdCl_2$ and 3 ml HCl were added to a 25 mL polytetrafluoroethylene lining and transferred to a stainless steel autoclave. The mixture solution was heated at 180 ℃ for 150 minutes, then cooled to room temperature. The samples were collected in a centrifugal tube and cleaned with absolute alcohol.

Synthesis of Mn^{2+}:PEA_2CdCl_4 SCs

Firstly, a certain concentration of Mn^{2+} ion-doped precursor solution was prepared. Mn^{2+} precursor solution with a molar concentration of 0.1 mmol/mL was obtained by dissolving 1 mmol $MnCl_2·4H_2O$ in 10 mL HCl. After that, Mn^{2+}:PEA_2CdCl_4 was synthesized by the same method as above. Different amounts of Mn^{2+} precursors were added while keeping the amount of $CdCl_2$ and $MnCl_2·4H_2O$ to 0.25 mmol.

Fabrication of LED Devices.

Firstly, the 15%Mn^{2+}:PEA_2CdCl_4 SCs were ground into powder in a mortar, then mixed with the UV-curable glue; the sample was coated on a small bulb with a 365 nm chip, and finally it was irradiation by UV lamp for 1 hour.

Preparation of flexible films

Firstly, the 15%Mn^{2+}:PEA_2CdCl_4 powder was mixed with Polydimethylsiloxane (PDMS) in a beaker. The mixture was stirred continuously for 60 minutes using a magnetic stirrer, then uniformly deposited onto a glass substrate. Finally, thermal curing was performed in a convection oven at 60 °C for 24 hours.

Characterizations

X-ray powder diffraction (XRD, Bruker D8 Discover) was employed to characterize the phase and crystal structure. X-ray photoelectron spectroscopy (XPS, Thermo Fisher Scientific ESCALAB250Xi) was utilized for identifying the elemental composition and chemical state. The Horiba Jobin Yvon Fluorolog-3 spectrometer was used to measure steady-state photoluminescence spectra, time-resolved spectra, temperature-dependent PL spectra and temperature-dependent time-resolved spectra. Absorption spectra were measured with the UV-VIS-NIR spectrophotometer (PerkinElmer Instruments, Lambda750). Device performance characterization was conducted using a white light LED detection system.

DFT Calculations

DFT calculations were conducted using the Vienna ab initio Simulation Package (VASP) code[1] with the

projection-augmented wave (PAW) method. The Perdew–Burke–Ernzerhof (PBE)[2] generalized gradient approximation (GGA) was employed as the exchange–correlation functional for structural relaxations and total-energy calculations of all structures. A cutoff energy of 350 eV and a convergence accuracy of 1 x 10^{-4} eV were used for the plane wave. The atomic stress convergence criterion for ion relaxation was set to be less than 0.005 eV/Å per atom. Data processing and graphical plotting were carried out using VESTA and Origin software.

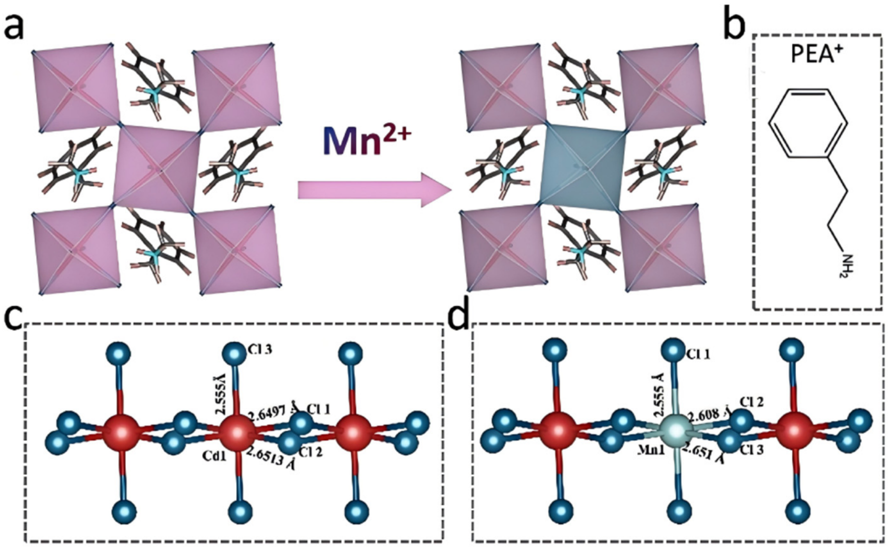

Figure S1 **(a)** Schematic diagram of organic amine insertion into inorganic sublattices in PEA_2CdCl_4 and Mn^{2+}:PEA_2CdCl_4 SCs; **(b)** the molecular formula of organic amine PEA; **(c)** A schematic diagram of $[CdCl_6]^{4-}$ octahedrons in PEA_2CdCl_4 and $[CdCl_6]^{4-}$ octahedrons substituted by $[MnCl_6]^{4-}$ in Mn^{2+}:PEA_2CdCl_4.

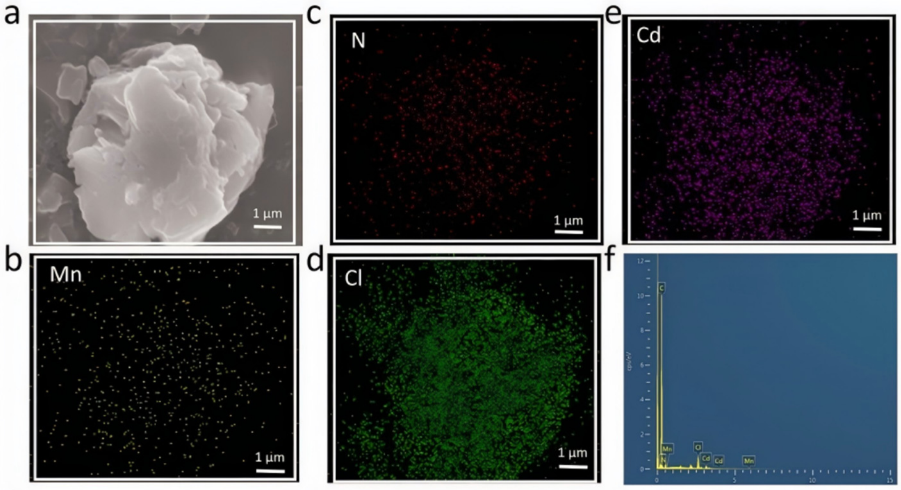

Figure S2 (a-d) The SEM image and EDS-mappings of N, Cd, Mn and Cl of 15% Mn^{2+}:PEA_2CdCl_4. **(f)** Energy-dispersed spectrum of 15% Mn^{2+}:PEA_2CdCl_4.

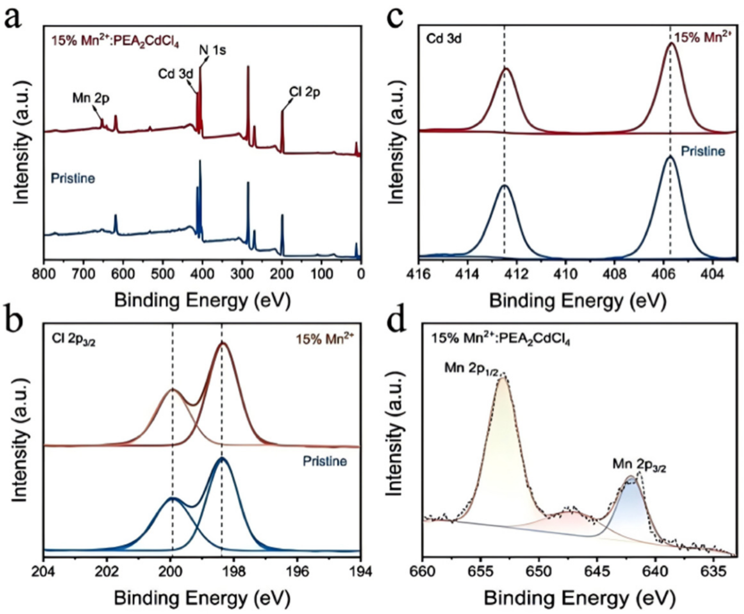

Figure S3 (a) XPS spectra of Mn^{2+}:PEA_2CdCl_4 and 15% Mn^{2+}:PEA_2CdCl_4. **(b-c)** High-resolution XPS spectra of Cd 3d and Cl 2p in PEA_2CdCl_4 and 15% Mn^{2+}:PEA_2CdCl_4. **(d)** High-resolution XPS spectra of Mn 2p in 15% Mn^{2+}:PEA_2CdCl_4.

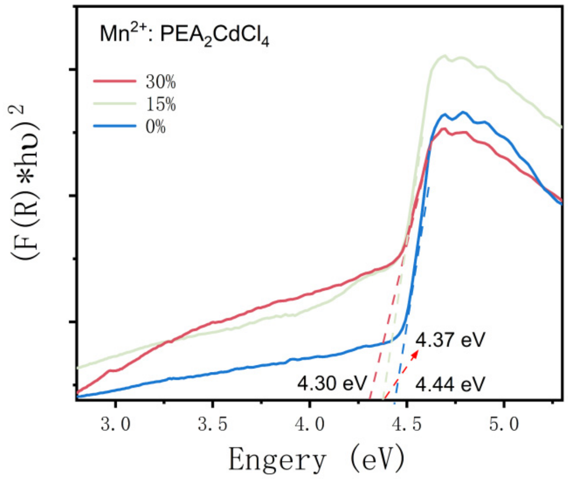

Figure S4 *x*% Mn^{2+}:PEA_2CdCl_4 (x = 0, 15, 30) band gaps calculated by using the K-M equation.

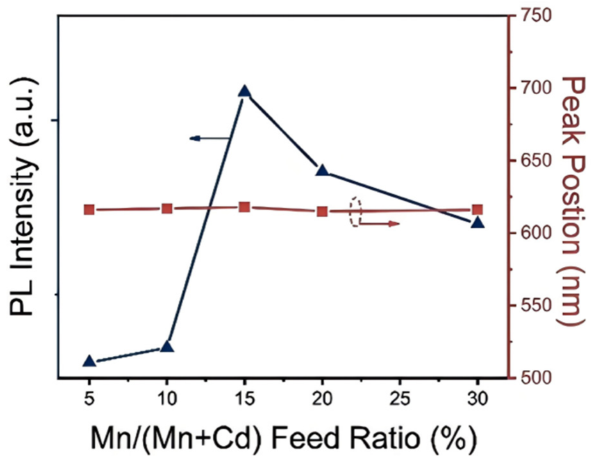

Figure S5 Changes of PL Intensity and peak position with Mn^{2+} doping concentration.

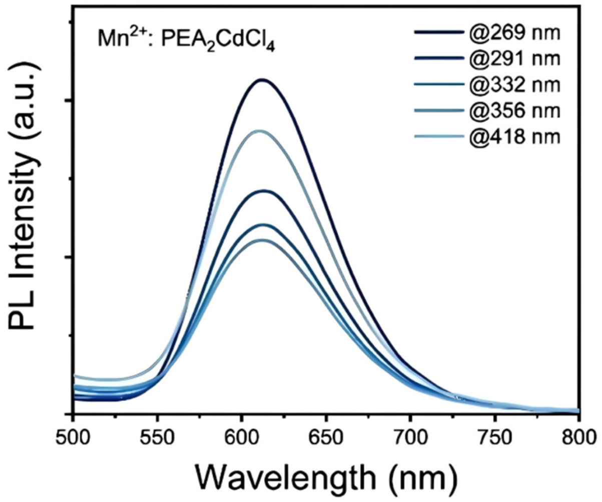

Figure S6 PL spectra under different excitations of 15%Mn^{2+}:PEA_2CdCl_4.

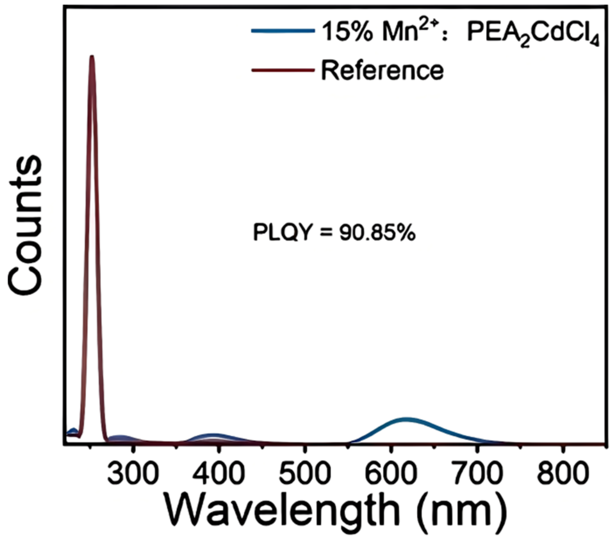

Figure S7. PLQY of 15%Mn^{2+}:PEA_2CdCl_4.

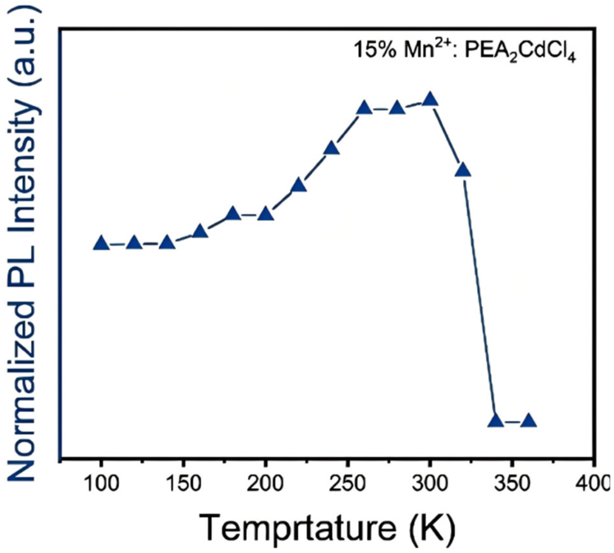

FigureS8 Temperature-dependent normalized PL intensity changes of 15%Mn^{2+}:PEA_2CdCl_4.

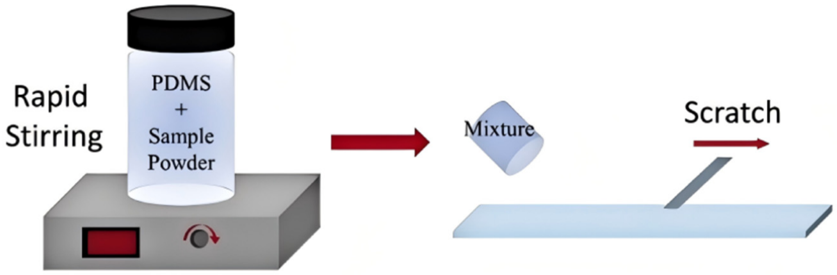

Figure S9 Schematic diagram of preparation of flexible film.
